# Preventing recurrence of endometriosis by means of long-acting progestogen therapy (PRE-EMPT): report of an internal pilot, multi-arm, randomised controlled trial incorporating flexible entry design and adaption of design based on feasibility of recruitment

**DOI:** 10.1186/s13063-017-1864-0

**Published:** 2017-03-11

**Authors:** Lee J. Middleton, Jane P. Daniels, Annalise Weckesser, Siladitya Bhattacharya, on behalf of the PRE-EMPT trial collaborative group, Tracy Roberts, Hilary O. D. Critchley, Christian Becker, Kevin Cooper, Janesh Gupta, Ertan Saridogan, Andrew Horne, Justin Clark, Andrew Prentice, Georgina Jones, Elaine Denny, Catherine Whittall

**Affiliations:** 1 Birmingham Clinical Trials Unit, Institute of Applied Health Research, College of Medical and Dental Sciences, University of Birmingham, Edgbaston, Birmingham, B15 2TT UK; 2 Centre for Health and Social Care Research, Birmingham City University, Birmingham, B15 3TN UK; 3 Institute of Applied Health Sciences, School of Medicine and Dentistry, Aberdeen Maternity Hospital, Foresterhill, Aberdeen, AB25 2ZD UK

**Keywords:** Endometriosis, LARC, Internal pilot, Multi-arm, Multi-stage, Flexible, Adaptation, Randomised trial

## Abstract

**Background:**

Endometriosis is associated with the growth of endometrium in ectopic sites mainly within the pelvis. This results in inflammation and scarring, causing pain and impaired quality of life. Endometriotic lesions can be excised or ablated surgically, but the risk of recurrence is high. A Heath Technology Assessment commissioning call in 2011 sought applications for trials aimed at evaluating long-term effectiveness of postoperative, long-acting, reversible contraceptives (LARCs) in preventing recurrence of endometriosis. A survey of gynaecologists indicated that there was no consensus about which LARC (Levonorgestrel Intrauterine System (LNG-IUS) or depot medroxyprogesterone acetate injection (DMPA)) or comparator (combined oral contraceptive pill (COCP) or no treatment) should be evaluated. Hence, we designed a ‘flexible-entry’ internal pilot to assess whether a four-arm trial was feasible including a possible design adaption based on pilot findings.

**Methods:**

In this pilot, women could be randomised to two, three or four treatment options provided that one was a LARC and one was a non-LARC. An assessment of feasibility based on recruitment to these options and a revised substantive trial design was considered by an independent oversight committee.

**Results:**

The study ran for 1 year from April 2014 and 77 women were randomised. Only 5 (6%) women accepted randomisation to all groups, with 63 (82%) having a LARC preference and 55 (71%) a non-LARC preference. Four-way and three-way designs were ruled out with a two-way LARC versus COCP design, stratified by prerandomisation choice of LARC and optional subrandomisation to LNG-IUS versus DMPA considered a feasible substantive study.

**Conclusions:**

Multi-arm studies are potentially efficient as they can answer multiple questions simultaneously but are difficult to recruit to if there are strong patient or clinician preferences. A flexible approach to randomisation in a pilot phase can be used to assess feasibility of such studies and modify a trial design based on chosen recruitment options, but trialists should consider carefully any practical arrangements should groups need to be dropped during a study.

**Trial registration:**

International Standard Randomised Controlled Trial Number, ISRCTN97865475. Registered on 20 March 2014.

**Electronic supplementary material:**

The online version of this article (doi:10.1186/s13063-017-1864-0) contains supplementary material, which is available to authorized users.

## Background

Endometriosis affects up to one in ten women, poses a considerable socioeconomic burden and has serious impact on quality of life. It is associated with growth of endometrium in abnormal locations such as the pelvic peritoneum, ovaries, fallopian tubes, bladder and bowel. These deposits undergo cyclical proliferation in response to ovarian hormones (mainly oestrogen) resulting in internal bleeding and inflammation, followed by scarring and adhesion formation. This results in pain, and has a profound negative impact on quality of life [1]. The ‘gold standard’ for the diagnosis of endometriosis is laparoscopy, a key-hole surgical procedure that allows direct visualisation and biopsy of endometriotic tissue. Surgical removal or destruction of endometriotic tissue is currently the preferred treatment for pain and other symptoms but relapse of symptoms occurs in 40–45% of women and 27% of women are readmitted for surgery within 5 years [2]. Half of all women diagnosed with endometriosis require a second operation and just over a quarter will undergo three or more procedures. Given the substantial cost, morbidity and prolonged recovery period associated with repeat surgery, there is an urgent need to identify an effective means of reducing the risk of symptom recurrence.

A 2004 Cochrane review [3] could not find sufficient evidence in favour of any medical treatment after conservative surgery. There were no long-term studies, with only some small trials considered to be lacking in quality or size to guide practice. In November 2011 the National Institute for Health Research Health Technology Assessment Programme (NIHR HTA) in the UK issued a commissioned call for trials evaluating ‘the clinical and cost-effectiveness of long-acting reversible contraceptives (LARCs) in preventing recurrence of endometriosis?’ LARCs are a group of hormonal treatments of particular interest as they are relatively cheap and have a prolonged duration of action, eliminating the need for daily administration, potentially improving patient compliance [4]. The patient group were to be women with endometriosis who had just been treated by surgical treatment with the main outcome of interest being the recurrence of symptoms in the long term (minimum duration of follow-up at least 3 years). The applicants were asked to justify the type of LARC that they thought most appropriate; the example options indicated included levonorgestrel intrauterine systems (LNG-IUS), a uterine insert than can last for up to 5 years, and depot medroxyprogesterone acetate injection (DMPA), an injection that needs to be administered every 3 months. Similarly, the applicants were asked to justify which ‘usual treatment’ should be used as the control comparator; e.g. the combined oral contraceptive pill (COCP). Gonadotropin-releasing hormone agonists (GNRHs) were not considered suitable for inclusion as they are not recommended for prolonged use due to risk of osteoporosis [5].

With the evidence base unable to guide the specifics of study design for a trial we turned to a survey of national practice in December 2011. Members of the British Society for Gynaecological Endoscopy were sent an online questionnaire asking (1) whether they prescribed postoperative hormonal treatments, (2) their most commonly used hormonal treatment and (3) the most relevant comparison for any future trial. Sixty-two members responded, with 56 of these having experience of treating endometriosis. Of these, 45 (80%) indicated that they prescribed hormonal treatments and 11 (20%) did not. GNRH, LNG-IUS, COCP, and DMPA were the most commonly used treatments but with none obviously preferred over the others (39, 38, 37 and 25 responses, respectively). Three comparisons of interest ranked higher than the others (40 responses): LNG-IUS versus no treatment (18, 45%); LNG-IUS versus COCP (17, 43%); and LNG-IUS versus DMPA (12, 30%), but again without any one particular favourite [6].

With no clear LARC or comparator favoured we needed to consider how two viable LARCs: LNG-IUS and DMPA, and two viable non-LARCs: COCP and no treatment could be accommodated into a substantive trial. A four-arm trial was the obvious choice but concerns were raised about whether patient views would prohibit recruitment to a design requiring consent to four different interventions – it was likely a large proportion of this population would have tried one or more of these treatments before as they are recommended by NICE for the initial (presurgical) management of endometriosis [7]. Pragmatic designs [8] where the patient or clinician could select their choice of LARC or non-LARC were considered, but concerns were raised about ultimately unsatisfying and underpowered comparisons. The non-LARC group was particularly problematic being a mixture of active (COCP) and nonactive (no treatment) options. Given these difficult design issues, we decided to use *feasibility of recruitment to a particular randomisation scheme* in an internal pilot phase of a larger study to guide the type of design which should be taken forward. Here, we report the design and findings of the internal pilot and the proposed design of the revised study.

## Methods

### Design

**Fig. 1:**
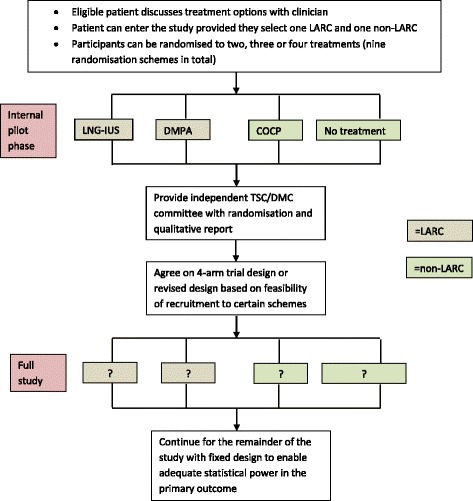
Study design incorporating flexible entry in an internal pilot phase and possible adaptation for substantive trial phase

Our proposal in the pilot phase was to include a flexible-entry design approach where participants could be randomised to two, three or four treatments provided that one was a LARC and the other was a non-LARC. This meant that a patient could enter any one of nine randomisation schemes (Fig. 1). On completion of the pilot phase a decision about the substantive study design based on feasibility of recruitment would be made. As no outcome assessment was proposed, inflation of type I error was not a concern [9]. The options for a definitive trial included continuing with a four-way randomisation design if acceptable to women, or alternatively to drop one or two treatment groups if randomisation proved difficult. The design would be fixed along with an appropriate sample size target to make sure that we would have enough power to detect a minimally important difference [10]. Data collected from participants randomised in the pilot phase to designs that remain in the main phase would be taken forward and combined with subsequent data collected; however, all women would be followed up to study conclusion regardless of randomisation options selected.

To ensure that an appropriate design choice was made, a post-pilot phase report was scheduled to be prepared for a joint Trial Steering Committee (TSC) – Data Monitoring Committee (DMC) meeting to review at the end of the pilot phase. The pilot phase was intended to last a year with a recruitment target of 100 participants. The TSC and DMC would have the final say on any proposed changes which would also be communicated to the funding body for approval. The post-pilot report was to include data on which randomisation schemes had been selected by patients and the results of a qualitative assessment (see below for methods). A threshold of any one group attracting less than 10% of the randomisations was set as providing evidence that it would not be feasible to include this group going forward. Any updated review of external evidence published since the grant application was submitted would also be taken into consideration. Apart from informing the design changes, the pilot phase also aimed to fine-tune operational procedures, assess data capture forms and confirm initial assumptions around sample size. The protocol is available at http://www.nets.nihr.ac.uk/projectsOld/hta/1111401.

### Population

Women aged 16–45 years with no immediate plan to conceive and scheduled to have laparoscopic treatment for pelvic pain associated with surgically confirmed endometriosis (or diagnostic laparoscopy with concurrent surgery if endometriosis found) were eligible. Women were not eligible if they were being treated for infertility, had plans for further elective endometriosis surgery, had any suspicion of malignancy or had contraindications to the use of hormonal treatment.

### Eligibility and randomisation

All women scheduled for surgery in the six participating centres were approached with information regarding the trial. They were provided with a Patient Information Sheet and given the opportunity to ask questions. Once eligibility was confirmed and treatment options had been discussed, the patient was asked for informed consent prior to surgery. Randomisation was undertaken intraoperatively, where feasible, to enable those allocated LNG-IUS to have it inserted at the end of the procedure under general anaesthetic or after conservative surgery but prior to discharge. A web-based central randomisation system at the University of Birmingham was used. A minimisation algorithm was used reflecting the desire to achieve balance with respect to a number of factors in the overall study. Stage of endometriosis (I to IV), extent of excision of endometriosis (complete or incomplete), age (below 35 years or 35 years and older) and recruiting centre were chosen as factors. A separate algorithm was used for each of the nine study entry options. Blinding of the patients and clinicians was not attempted as it was felt that it was not ethically justifiable due to the differing nature of the interventions.

### Procedures

Administration of the allocated medical treatment depended on local policy; ideally this was to be initiated prior to discharge. Subsequent injections for DMPA or prescriptions for COCP were to be delivered by the participant’s GP. LNG-IUS could be inserted by the woman’s GP if not fitted at the time of operation. Those allocated no treatment were asked to use barrier contraceptive methods if required. Further details are available in the protocol.

### Sample size and statistics

The primary outcome measure for the substantive trial was proposed to be the pain domain of the Endometriosis Health Profile (EHP)-30 questionnaire [11] at 3 years’ follow-up. The sample size for the substantive study conservatively assumed to be taking forward a four-way design and was powered (80%) to detect a 10-point difference on the EHP-30, assuming the standard deviation (SD) to be 25 points and type I error to be *p* = 0.01 (chosen through Bonferroni correction – albeit rounded to two decimal places – to allow for six potential comparisons, i.e. all groups compared against each other). This requires 592 patients, increased to 750 to allow for the possibility of 20% loss to follow-up. No hypothesis testing or formal analysis was planned for the dissemination of the internal pilot findings, with the primary aim to assess feasibility.

### Qualitative assessment

A qualitative assessment of barriers and facilitators to the recruitment was conducted at three of the recruiting centres. One focus group and ten individual semistructured interviews were conducted in order to elicit women’s past experiences with the proposed treatments and to assess whether they constituted a barrier to participation. The focus group discussion took place in one of the centres and included four women. Three women were interviewed in their homes and seven were interviewed over the telephone. The focus group and interviews were recorded and transcribed verbatim. Content analysis [12] was employed with a qualitative lead and two assistants independently reading the transcripts and agreeing upon common themes. Dissident views were also considered. Further details are described in the protocol.

## Results

### Recruitment

Six centres in the UK were involved in the pilot trial with staggered starts from April 2014 to the end of March 2015 (recruiting on average for 10.5 months). During this period 504 patients were assessed for eligibility with 77 patients recruited. The most common reasons for ineligibility were: plan to conceive in the near future (42 patients, 10%); contraindications to one or more treatments (35 patients, 8%); no endometriosis identified at diagnostic laparoscopy (33 patients, 8%). The main reason for not wanting to take part was because of a preference for a particular treatment (94 patients, 22%; the most common LNG-IUS, 30 and DMPA, 30). Details of randomised participants are given in Table 1. The majority (56/77, 73%) had minimal to mild disease and complete excision (71/77, 92%) as judged by the operating surgeon. The average participant rated endometriosis-related pain to be 58 (SD 19) out of 100 (worst score) as measured by the EHP-30.

**Table 1:** Details of participants included in the pilot phase

		*n* = 77
Age, years	Mean (SD)	31 (7.5)
Age <35 years, *n* (%)	Yes	53 (69)
	Missing	0
BMI, kg/m^2^	Mean (SD)	27 (5.7)
	Missing	17
Ethnic group, *n* (%)	White British	64 (86)
	Black/Black British Caribbean	2 (3)
	Asian/Asian British Indian	3 (4)
	Asian/Asian British Pakistani	1 (1)
	Mixed White/Black Caribbean	2 (3)
	Mixed White/Asian	1 (1)
	Other mixed background	1 (1)
	Missing	3
Stage of endometriosis, *n* (%)	I	36 (47)
	II	20 (26)
	III	11 (14)
	IV	10 (13)
	Missing	0
Ever smoked? *n* (%)	Yes	34 (48)
	Missing	6
Extent of excision as judged by surgeon, *n* (%)	Complete	71 (92)
	Missing	0
EHP-30 pain score	Mean (SD)	58 (18.5)
	Missing	2

*BMI* Body Mass Index, *EHP* Endometriosis Health Profile, *SD* standard deviation

### Randomisation options chosen

**Fig. 2:**
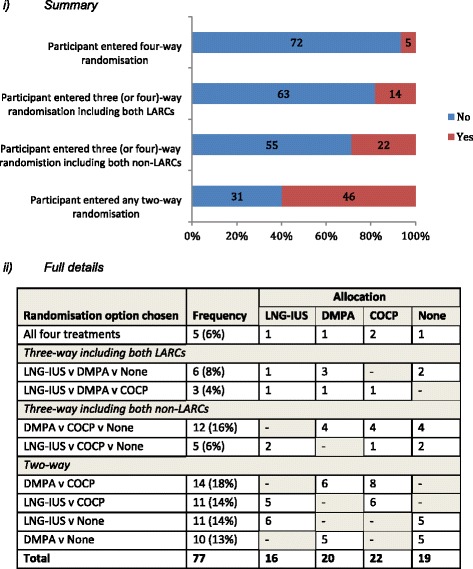
Randomisation options chosen

Only 5 of the 77 participants (6%) were willing to be randomised to all four treatment options (Fig. 2). Participants willing to be randomised to both LNG-IUS and DMPA were relatively low, with the vast majority, 82% (63/77), expressing a preference for one or the other in roughly even proportions (43% for LNG-IUS and 57% for DMPA). In a similar fashion, most, 71% (55/77) expressed a preference for their choice of comparator in even proportions (51% for COCP and 49% for no treatment). Forty-six of the participants (60%) expressed a preference for both a LARC and their comparator and hence opted for variations of two-way randomisations.

### Qualitative assessment

All women who agreed to participate in the qualitative element were included in the pilot trial. They represented a range of symptomatology, treatment histories and allocated trial treatment groups. Women found flexible randomisation acceptable as they had an element of choice over which treatment groups to be randomised to. Half of participants (*n* = 7) reported that without the option to opt out of a particular treatment group (or groups) they would have declined trial participation. No single treatment group was found more or less acceptable to women. Women made decisions regarding which treatment arms were acceptable based on their past negative or positive treatment experiences and the treatment experiences of significant others (female friends and family). Women chose to participate in the trial for reasons of altruism and self-interest and found the 3-year length of their participation acceptable [13].

### New external evidence

Since the initial study proposal, two systematic reviews of variable quality have examined the use of COCP in this population. The first [14] identified 15 randomised trials including 850 patients. The combined odds of recurrence was noted to be lower in the COCP group compared with surgery alone (odds ratio (OR) 0.31, 95% CI 0.22, 0.45; *p* < 0.001) The second [15] evaluated the use of prolonged (at least 2 years) postoperative COCP use on endometrioma recurrence in a total of 965 women (726 in cohort studies and 239 in one randomised controlled trial). Recurrence was lower with COCP compared with no treatment (OR 0.12; 95% CI 0.05–0.29; *p* < 0.001).

### Proposal for adapted trial design

**Fig. 3:**
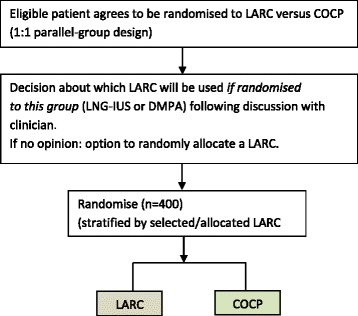
Revised trial design for substantive study

Four-way and three-way randomisation designs were ruled out due to low numbers selecting these randomisation options. We also ruled out a trial design involving solely the most commonly selected two-way randomisation option (DMPA versus COCP) as this attracted only 14 participants (18% of all participants). Given the strong preferences noted (including in the qualitative work) we decided to incorporate some element of choice in the revised design (Fig. 3). The main comparison proposed was LARC, considered as a class of treatments, versus COCP, with LARC selected prerandomisation by the patient if a preference was apparent (or alternatively allocated randomly if there was no opinion). The choice of LARC would need to be decided prior to randomisation to enable unbiased stratified (subgroup) analyses of LNG-IUS versus COCP and DMPA versus COCP (e.g. only those selecting LNG-IUS prerandomisation would be included in a LNG-IUS versus COCP comparison). COCP was chosen as the comparator over no treatment on the basis of the new external evidence.

The sample size was revised to reflect a main two-arm comparison and took into consideration a revised estimate of the SD of the primary outcome (from pooled baseline data). To detect an 8-point difference on the EHP-30 pain domain with 90% power (*p* = 0.05) and assuming the SD is 22 points requires 160 participants per group, 320 in total (to account for any loss to follow-up – estimated 20% – this target was inflated to 400). Eight points is equivalent to 0.36 SD, which can be considered half-way between a small (0.2 SD) to moderate (0.5 SD) effect size [16]. This size of sample would also give us good power (80%) to detect a 10-point difference in the two stratified analyses of LNG-IUS versus COCP and DMPA versus COCP provided the remaining recruits into the study have a roughly even split. Our estimates from the pilot suggest that only a minority of patients would enter the LNG-IUS versus DMPA comparison but, nevertheless, some randomised data would be better than nothing as none currently exist. This design was ratified by the external DMC-TSC committee in April 2015 and subsequently approved by the funding body.

## Discussion

In this flexible internal pilot study of medical treatments following surgery for endometriosis, we found that a very low proportion of the participants were willing to be randomised to all four treatment options on offer; indeed most were only willing to be randomised to two treatments that did not include both long-acting contraceptives. Qualitative assessment found that women were receptive to having some element of control over which groups they were to be randomised to. No one particular treatment was favoured and patient decisions were based on previous experiences with these treatments. Meanwhile, emerging evidence suggests that COCP is more effective for prevention of recurrence of pain following surgery for endometriosis than no treatment in this population. Given these findings, we revised our study to include a main comparison of LARC versus COCP, with LARC preselected ahead of randomisation to also enable stratified analysis of DMPA versus COCP and LNG-IUS versus COCP.

Where multiple treatment options are possible, a flexible approach to randomisation in an internal pilot is one that could be applicable in settings outside of gynaecology. Flexible trial designs have proved beneficial in other trial situations [17, 18], allowing multiple questions to be simultaneously addressed, but often the flexibility is provided to accommodate clinician preferences or practice, rather than those of the participants. The advantage of this approach is that it allowed the trial team to engage with, and listen to, patients faced with real-life decisions regarding randomisation (as opposed to data from a survey of potential participants). An easier choice would have been to plan a two-group trial which could have limited the number of questions that could be potentially answered or, alternatively, to embark on a four-arm trial that was incapable of recruiting. The assessment of the randomisation data was preplanned and was overseen and approved by an external, independent committee including expert clinical and statistical advisors. We believe our revised design to be feasible and it enables us to include three of the four options initially identified and at the same to incorporate some element of patient choice which was very apparent. We also hope that this element of patient choice will result in faster recruitment than other design alternatives but accept that this will be not be possible to prove conclusively.

We underestimated the strength of opinion for certain treatments and were wrong in predicting that some would show obviously poorer recruitment than others, allowing these to be confidently dropped and leading to a ‘neat’ substantive trial design. Reality was more rather more complicated and gave us results of preference that were less straightforward. The independent TSC/DMC was important in this respect to make sure that we retained a study capable of changing practice rather than one that was just ‘easy to answer’. The decision to combine two different interventions (LNG-IUS and DMPA) into one LARC drug class for the main trial was a difficult one as, although similar pharmacologically (progestogen), they have very different routes of administration. However, this was considered by the committee members to be a pragmatic response to the pilot evidence outlined above.

We could be criticised for not setting more stringent decision rules for all of the numerous randomisation scenarios; this is something that should be given further consideration in other studies wishing to adopt this approach. Further criticism of our approach may be that we have actually encouraged patient preference by offering it; we cannot answer whether this is indeed true from this research. Incorporating patient preference is controversial but some have argued that it can increase the generalisability of study findings [19].

Whilst other trialists have reported positive experiences with multi-arm, multi-stage trials [20] their practical challenges should not be underestimated if one or more groups need to be stopped mid trial. We found adaptations to be logistically difficult and took much longer than we anticipated; 3 months were planned for study revisions and in reality this was closer to 9 months. The funding body considered further peer review necessary and we were also required to revise costs in line with the reduced sample size. In addition, changes to the case report forms, protocol, database, Research Ethics Committee (REC) approvals, retraining for site randomisers, etc. have all taken considerable work. Study designs such as this or adaptive designs [21], whilst theoretically efficient on one hand, also have the potential for waste in this respect; processes for rapid implementation of changed designs should be considered from the outset to mitigate against any potential delays [22].

Endometriosis is a difficult disease to study with issues around diagnosis, reproductive intentions of the participants and suitability of outcomes [23]. There is an urgent need for research into endometriosis, with serious gaps in the current evidence base [3]. This is the only study registered on the WHO Trial Portal (accessed on 18 May 2016) to our knowledge researching whether LARCs are the best way to treat women following surgery to prevent recurrence of symptoms. Our findings suggest that research in this field is likely to remain challenging with some questions, such as which LARC is more effective (LNG-IUS or DMPA), likely to prove difficult to answer due to the strength of patient opinions.

## Conclusions

There is an obvious need for large publically funded trials to have feasibility assessments incorporated into their design. If more than two interventions are on offer, trialists have the dilemma of whether to embark on a multi-arm study, which may increase the difficulty of recruitment, or to revert to the ease of a two-group study. In this respect, our approach to assessing where a multi-arm study is feasible and, if not, to design a feasible study going forward is a novel one and may influence the design of future studies.

### Trial status

PRE-EMPT is currently recruiting into the trial with the revised study design.

## Additional file

Additional file 1: CONSORT flow diagram and Checklist. (DOCX 34 kb)
