# Supplementary material for: Preventing recurrence of endometriosis by means of long-acting progestogen therapy (PRE-EMPT): report of an internal pilot, multi-arm, randomised controlled trial incorporating flexible entry design and adaption of design based on feasibility of recruitment
Source: Trials. 2017 Mar 11;18:121. doi: 10.1186/s13063-017-1864-0 (PMC5346193; doi:10.1186/s13063-017-1864-0)
Supplement: Additional file 1: — CONSORT flow diagram and Checklist. (DOCX 34 kb) [file 13063_2017_1864_MOESM1_ESM.docx]

Supplementary appendix A – CONSORT flow diagram (completed where applicable to internal pilot)

504 women screened

127 ineligible

- Plans to conceive in the immediate future n=42
- Contradictions to one or more treatments n=35
- No endometriosis identified n=33
- Other inclusion/exclusion criteria n=17

94 declined with treatment preference

- Patient wants DPMA n=30
- Patient wants LNG-IUS n=30
- Preferred choice of other treatment n=22
- Patients wants COCP n=6
- Does not want LNG-IUS or DMPA n=6

66 other reason

140 no reason given

77 randomised

16 allocated LNG-IUS

19 allocated no treatment

20 allocated DMPA

22 allocated COCP

Supplementary appendix B – CONSORT checklist (completed where applicable to internal pilot)

| **Section/Topic** | **Item No** | **Checklist item** | **Reported on page No** |
| --- | --- | --- | --- |
| **Title and abstract** | | | |
|  | 1a | Identification as a randomised trial in the title | 1 |
|  | 1b | Structured summary of trial design, methods, results, and conclusions (for specific guidance see CONSORT for abstracts) | 2-3 |
| **Introduction** | | | |
| Background and objectives | 2a | Scientific background and explanation of rationale | 4-6 |
|  | 2b | Specific objectives or hypotheses | 6 |
| **Methods** | | | |
| Trial design | 3a | Description of trial design (such as parallel, factorial) including allocation ratio | 6 |
|  | 3b | Important changes to methods after trial commencement (such as eligibility criteria), with reasons | n/a |
| Participants | 4a | Eligibility criteria for participants | 7 |
|  | 4b | Settings and locations where the data were collected | 7 |
| Interventions | 5 | The interventions for each group with sufficient details to allow replication, including how and when they were actually administered | 8 |
| Outcomes | 6a | Completely defined pre-specified primary and secondary outcome measures, including how and when they were assessed | 8 |
|  | 6b | Any changes to trial outcomes after the trial commenced, with reasons | n/a |
| Sample size | 7a | How sample size was determined | 8 |
|  | 7b | When applicable, explanation of any interim analyses and stopping guidelines | 6-7 |
| Randomisation: |  |  |  |
| Sequence generation | 8a | Method used to generate the random allocation sequence | 8 |
|  | 8b | Type of randomisation; details of any restriction (such as blocking and block size) | 8 |
| Allocation concealment mechanism | 9 | Mechanism used to implement the random allocation sequence (such as sequentially numbered containers), describing any steps taken to conceal the sequence until interventions were assigned | 8 |
| Implementation | 10 | Who generated the random allocation sequence, who enrolled participants, and who assigned participants to interventions | 7-8 |
| Blinding | 11a | If done, who was blinded after assignment to interventions (for example, participants, care providers, those assessing outcomes) and how | n/a |
|  | 11b | If relevant, description of the similarity of interventions | n/a |
| Statistical methods | 12a | Statistical methods used to compare groups for primary and secondary outcomes | n/a (as internal pilot) |
|  | 12b | Methods for additional analyses, such as subgroup analyses and adjusted analyses | n/a (as internal pilot) |
| **Results** | | | |
| Participant flow (a diagram is strongly recommended) | 13a | For each group, the numbers of participants who were randomly assigned, received intended treatment, and were analysed for the primary outcome | Figure 2 (partial as internal pilot) |
|  | 13b | For each group, losses and exclusions after randomisation, together with reasons | n/a (as internal pilot) |
| Recruitment | 14a | Dates defining the periods of recruitment and follow-up | 9 (partial as internal pilot) |
|  | 14b | Why the trial ended or was stopped | n/a (as internal pilot) |
| Baseline data | 15 | A table showing baseline demographic and clinical characteristics for each group | Table 1 (partial as internal pilot) |
| Numbers analysed | 16 | For each group, number of participants (denominator) included in each analysis and whether the analysis was by original assigned groups | n/a (as internal pilot) |
| Outcomes and estimation | 17a | For each primary and secondary outcome, results for each group, and the estimated effect size and its precision (such as 95% confidence interval) | n/a (as internal pilot) |
|  | 17b | For binary outcomes, presentation of both absolute and relative effect sizes is recommended | n/a (as internal pilot) |
| Ancillary analyses | 18 | Results of any other analyses performed, including subgroup analyses and adjusted analyses, distinguishing pre-specified from exploratory | n/a (as internal pilot) |
| Harms | 19 | All important harms or unintended effects in each group (for specific guidance see CONSORT for harms) | n/a (as internal pilot) |
| **Discussion** | | | |
| Limitations | 20 | Trial limitations, addressing sources of potential bias, imprecision, and, if relevant, multiplicity of analyses | n/a (as internal pilot) |
| Generalisability | 21 | Generalisability (external validity, applicability) of the trial findings | n/a (as internal pilot) |
| Interpretation | 22 | Interpretation consistent with results, balancing benefits and harms, and considering other relevant evidence | n/a (as internal pilot) |
| **Other information** | | |  |
| Registration | 23 | Registration number and name of trial registry | Page 3 |
| Protocol | 24 | Where the full trial protocol can be accessed, if available | 7 |
| Funding | 25 | Sources of funding and other support (such as supply of drugs), role of funders | 15 |
